# Microdialysis shows metabolic effects in skin during fluid resuscitation in burn-injured patients

**DOI:** 10.1186/cc5124

**Published:** 2006-12-13

**Authors:** Anders Samuelsson, Ingrid Steinvall, Folke Sjöberg

**Affiliations:** 1 Department of Intensive Care, Linköping University Hospital, 581 85 Linköping, Sweden; 2 The Burn Unit, Department of Hand and Plastic Surgery, Linköping University Hospital, 581 85 Linköping, Sweden; 3 Faculty of Health Sciences, Department of Biomedicine and Surgery, Linköping University Hospital, 581 85 Linköping, Sweden

## Abstract

**Introduction:**

Established fluid treatment formulas for burn injuries have been challenged as studies have shown the presence of tissue hypoxia during standard resuscitation. Such findings suggest monitoring at the tissue level. This study was performed in patients with major burn injuries to evaluate the microdialysis technique for the continuous assessment of skin metabolic changes during fluid resuscitation and up to four days postburn.

**Methods:**

We conducted an experimental study in patients with a burn injury, as represented by percentage of total body surface area burned (TBSA), of more than 25% in a university eight-bed burns intensive care unit serving about 3.5 million inhabitants. Six patients with a median TBSA percentage of 59% (range 33.5% to 90%) and nine healthy controls were examined by intracutaneous MD, in which recordings of glucose, pyruvate, lactate, glycerol, and urea were performed.

**Results:**

Blood glucose concentration peaked on day two at 9.8 mmol/l (6.8 to 14.0) (median and range) and gradually declined on days three and four, whereas skin glucose in MD continued to increase throughout the study period with maximum values on day four, 8.7 mmol/l (4.9 to 11.0). Controls had significantly lower skin glucose values compared with burn patients, 3.1 mmol/l (1.5 to 4.6) (*p *< 0.001). Lactate from burn patients was significantly higher than controls in both injured and uninjured skin (MD), 4.6 mmol/l (1.3 to 8.9) and 3.8 mmol/l (1.6 to 7.5), respectively (*p *< 0.01). The skin lactate/pyruvate ratio (MD) was significantly increased in burn patients on all days (*p *< 0.001). Skin glycerol (MD) was significantly increased at days three and four in burn patients compared with controls (*p *< 0.01).

**Conclusion:**

Despite a strategy that fulfilled conventional goals for resuscitation, there were increased lactate/pyruvate ratios, indicative of local acidosis. A corresponding finding was not recorded systemically. We conclude that MD is a promising tool for depicting local metabolic processes that are not fully appreciated when examined systemically. Because the local response in glucose, lactate, and pyruvate metabolism seems to differ from that recorded systemically, this technique may offer a new method of monitoring organs.

## Introduction

Severe burns result in both local and systemic responses. There is loss of homeostatic control as a result of massive losses of fluid and protein during the first 24 hours. This is usually followed by a normalisation of permeability and reduced fluid losses during the second day [1,2]. To counteract this first phase, resuscitation aims to replace lost fluid. The massive amount of fluid needed during resuscitation, particularly in larger burns, creates a generalised oedema that is caused both by the volume of fluid itself and the decreased colloid osmotic pressure that will ensue secondary to the resuscitation fluid given and to proteins lost from the circulation [3,4]. This may compromise tissue perfusion in both injured and uninjured tissues of the burn-injured patient.

The burn also elicits the general trauma response, including the increase in blood glucose concentrations as a result of glyconeogenesis, glycogenolysis, insulin-resistance, and lipolysis. There is also catabolism of lean body mass that involves the metabolism of protein [5,6]. These changes have often been studied systemically, even in humans, but less is known about the changes in injured tissues.

Over the years, several regimens have been used for resuscitation [7-9]. The Parkland formula, 2 to 4 ml/kg × total body surface area burned (TBSA) percentage per 24 hours, is the most widely used [10,11]. It is designed to ensure perfusion of organs and tissues and is aimed at avoiding overhydration. The resuscitation strategies are generally guided by blunt endpoints such as urinary output (0.5 to 1 ml/kg per hour) and mean arterial pressure (more than 70 mm Hg). However, most of the current regimens for resuscitation of burned patients may be inadequate to produce both optimal central haemodynamics and ideal conditions at the organ and tissue levels [12,13]. Present research that is designed to improve resuscitation formulas has included both the need for more fluid by investigators who have looked mainly at the central circulations [13,14] and the need for less fluid or the use of colloids by others who have the needs of the tissues in mind [8,9]. Lately, there seems to be a tendency to increase the volume of fluid given to burned patients [15]. Severe burns are often complicated by multiple-organ failure, indicating that current resuscitation strategies and endpoints may be inadequate in that they produce regions of tissue hypoxia and ischaemia [16,17]. This emphasises the need for more specific endpoints that focus on the tissue perspective in injured and in uninjured tissues [18,19].

Microdialysis is an interesting technique for *in vivo *sampling of extracellular fluid. It can be applied adjacent to an injury or in the tissue at the site of an injury [20]. The method was originally designed for use in experimental studies of the brain in animals and focused on neurotransmitters [20] but has been developed and has become used extensively for metabolic studies in human skin, mostly experimentally [21,22], but also to follow blood flow and metabolic changes during exercise and critical limb ischaemia [23] and to study the metabolism of adipose tissue in patients in intensive care [24]. However, to our knowledge, the method has never been used in patients with burns, although the organ of interest (the skin) is easily accessible. It has been used to study burns in animals in which histamine turnover in the skin was examined successfully [25]. More recently, it has been used in studies of skeletal muscle and brain metabolism for the prediction of ischaemia and changes in the metabolism of glucose [20,26]. It has also been used to assess the metabolism, permeability, and local inflammation of skin in dermatology [27].

The aim of the present study was to assess metabolic events in the skin in patients with burns (local tissue changes in glucose, lactate, pyruvate, lactate/pyruvate ratio, glycerol, and urea) by using microdialysis during the course of conventional fluid resuscitation. We also investigated the metabolism of both injured (superficial second-degree burn) and uninjured tissues in burned patients and compared them with the metabolism of the skin in healthy controls.

## Materials and methods

After obtaining ethical committee approval and informed consent from patients or close relatives, we studied five men and one woman (median age 27.5 years [range 17 to 31] and median TBSA percentage of 59% [33.5% to 90%]). Four of the patients had flame burns and two had full-thickness chemical burns (Table 1). Age-matched healthy hospital staff and medical students (*n *= 9) acted as controls.

### Treatment protocol

Oxygen was given to maintain an SaO_2 _(arterial oxygen saturation) of more than 90%, and central venous and intra-arterial lines were inserted. The size of the burn was assessed using the Lund and Browder diagram. Patients were bronchoscoped to diagnose inhalation injury. Initial fluid resuscitation was given based on the Parkland formula (2 to 4 ml/kg × TBSA percentage per 24 hours) and was adjusted to maintain a urinary output of more than 0.5 ml/kg per hour during the first 24 hours. Mean arterial pressure (MAP) of more than 70 mm Hg served as a secondary endpoint. Colloids were withheld during the first 18 hours and were then given as albumin 5% or 20% or as hexastarch 10% (HAES; Fresenius Kabi AG, Bad Homburg, Germany) when there was circulatory instability. Patients were fed enterally as soon as possible (Nutrison; Nutricia Nordica AB, Stockholm, Sweden), starting at 10 ml/hour on day one and thereafter increasing daily until the nutritional goal was achieved. Patients received a glucose infusion of 2,000 ml/200 g per 24 hours starting on day two. The nutritional goal was to reach 25 kcal/kg per 24 hours in three to five days. Insulin was not provided during the study period. If the patient did not tolerate enteral nutrition, total parenteral nutrition (Vitrimix; Fresenius Kabi AB, Uppsala, Sweden) was provided. Blood transfusions were given to maintain a haemoglobin concentration above 9 g/dl. Plasma was provided if there were signs of excessive bleeding judged to have resulted from a lack of coagulation factors. All burns were excised and grafted for the first time within 36 hours. All burn-related data were prospectively recorded in the burns unit database [28]. Blood gases (i-STAT; i-STAT Corporation, East Windsor, NJ, USA) and blood glucose (HemoCue AB, Ängelholm, Sweden) were obtained four times per 24 hours and were analysed bedside. All other blood samples were obtained according to a set protocol and analysed at the Department of Clinical Chemistry at the Linköping University Hospital.

### Microdialysis

After they had been informed about the research procedures and had given their consent, the patients were examined and an area with deep second-degree (partial-thickness) burns on the trunk or proximal limb was chosen for the microdialysis experiments. The second-degree burn was defined as an area that maintained sensitivity to skin prick and that bled slightly at the site of needle punctures. The area was then disinfected with chlorhexidine in alcohol (Klorhexidine^® ^5 mg/ml, Fresenius Kabi AS, Halden, Norway). A venous cannula (1.4 mm, outer diameter) was inserted intradermally, and the position was accepted if the whole metal stylet could be seen through the skin. The metal stylet was withdrawn and the plastic tubing was cut 1 to 2 cm from the skin. The microdialysis catheter (membrane, 10 mm long; cutoff, 20,000 Da) (CMA 70; CMA Microdialysis AB, Solna, Sweden) was inserted through the plastic tubing of the venous cannula, which then was withdrawn. With the same technique, a second catheter was inserted into uninjured skin 5 to 10 cm away from the first catheter. A 1-ml microsyringe was fitted to a precision pump (CMA 102; CMA Microdialysis AB) and connected to the catheter tubing, and the system was perfused with lactate-free Ringer solution (CMA perfusion fluid; Na 147, K 4, Ca 2.3, and Cl 156 mmol/l; CMA Microdialysis AB). The probes were perfused at a rate of 0.5 μl/minute. The perfusate was collected in microvials that were capped to avoid evaporation of fluid and was kept on ice in the dark. If the yield of fluid slowed, the complete system (including catheters) was replaced, and the new catheter was placed as close as possible to the previous site and in the same blister or an adjacent blister within the stated area (that is, within 5 to 10 cm). A total of four catheters were replaced because they dislocated accidentally, probably due to high interstitial pressure. Recording was restarted after a three hour equilibration period. Sampling was continued until the patient started to mobilise (usually at day five). Interruptions were inevitable during operations. The perfusate was collected every third hour. Sampled vials were immediately frozen (-20°C) and stored in the freezer until analysis. All samples were analysed within three months. Analysis of the perfusate for glucose, urea, glycerol, lactate, and pyruvate was performed by a photometric assay in a fully automated analyser (CMA 600 Microdialysis Analyser; CMA Microdialysis AB).

The age-matched controls were given CMA 70 microdialysis catheters identical to those used for the patients, but we used a different, portable pump, the CMA 107 (CMA Microdialysis AB). The catheters were placed intracutaneously in the abdomen at the umbilical level. Samples of microdialysis fluid were collected every third hour, except at night, when the controls were instructed to change vials when they went to bed and again when they woke up. They were asked to avoid strenuous physical exercise, but no other restrictions in daily life were imposed. Sampling continued for three consecutive days. The perfusate was handled and analysed in the same way as for the patients.

### Data and statistical analysis

Data are presented as median (range) and are shown as box-and-whisker plots (median, with 25/75 and 10/90 percentiles). Medians were chosen because the data often showed a skewed distribution. Outliers in the graphs are values between 1.5 and 3 times the height of the box, above or below. Extremes are values more than three times the height of the box. Data from days two to four were used in all analyses. Because more than one sample a day was obtained, median values for each day were calculated and used in the analyses. The nine controls generated a total of 30 microdialysis values, which were examined for time-dependent changes, but because we found none, the mean value per control was calculated and these values were analysed as a group. To evaluate differences between controls and patients, we used the Mann-Whitney *U *test; we used the Bonferroni correction factor for multiple comparisons. Furthermore, because we were unable to find any differences in the microdialysis data between uninjured and burn-injured tissue, the tissue data were also analysed as a group. Correlations between skin glucose levels and lactate, pyruvate, and lactate/pyruvate quotients were performed using Spearman rank correlation. All statistical analyses were performed using Statistica, version 7.0 (StatSoft, Inc., Tulsa, OK, USA). Probabilities of less than 0.05 were considered significant.

## Results

The patients were given Ringer's acetate 3.6 ml/kg (2.1 to 5.9) × TBSA percentage. Urinary output was 1.99 ml/kg per hour (1.4 to 2.2) and MAP was 76 mm Hg (70 to 95) on day one. For the remaining three days of the study, urinary output was 1.0 ml/kg per hour (0.7 to 1.9). MAP was 76 mm Hg (60 to 95).

### Blood analyses

The concentration of glucose in blood increased from 8.0 mmol/l (7.0 to 9.0) on day one and reached a maximum of 9.8 mmol/l (6.8 to 14.0) on day two. There was a gradual reduction on days three and four (6.6 mmol/l [4.3 to 13.3] on day four). There were no signs of systemic acidosis during the study period (days two to four). Median arterial blood pH, base excess, and pCO_2 _(partial pressure of carbon dioxide) were within the reference ranges: 7.48 (7.38 to 7.58), 4.6 (-0.3 to 7.7), and 5.2 kPa (3.2 to 6.6), respectively. None of the patients had signs of renal failure; blood urea nitrogen was 4.0 mmol/l (1.9 to 10.1).

### Microdialysis

#### Glucose

**Figure 1:**
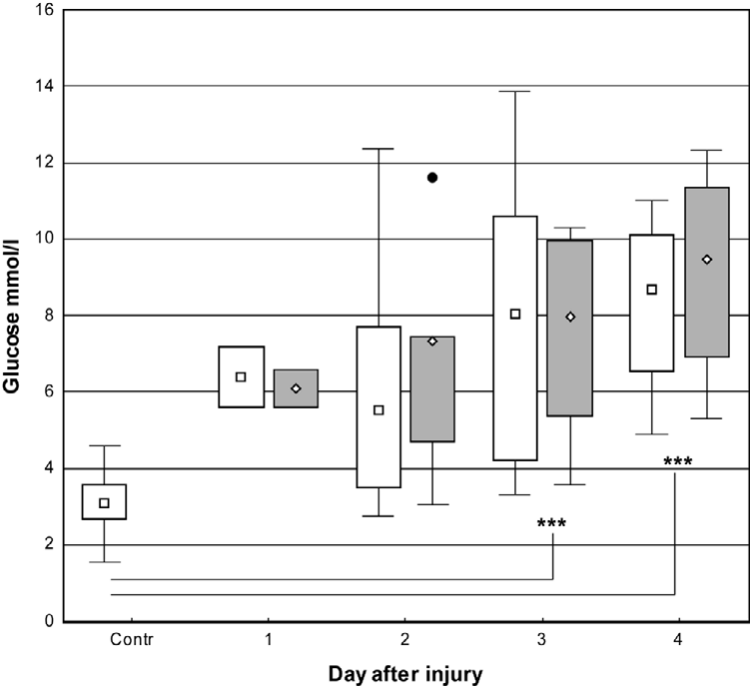
Box-and-whisker plots showing median (interquartile) glucose concentrations in microdialysate from days one to four. Open boxes indicate uninjured skin and controls; shaded boxes indicate burned skin. Filled circle indicates outlier (burned skin). Controls, *n *= 9. Uninjured skin on day 1, *n *= 2; day 2, *n *= 5; day 3, *n *= 6; and day 4, *n *= 5. Burned skin on day 1, *n *= 2; day 2, *n *= 5; day 3, *n *= 6; and day 4, *n *= 4. ***P < 0.001. Contr, control.

The cutaneous concentration of glucose increased in parallel to that in blood but, unlike blood concentrations, did not peak on day two. Instead, it continued to increase throughout the study period, reaching maximum values of 8.7 mmol/l (4.9 to 11.0) on day four in uninjured skin and 8.5 mmol/l (5.2 to 12.3) in injured skin. Cutaneous glucose concentrations in controls (3.1 mmol/l [1.5 to 4.6]) were significantly lower than in burned patients (*p *< 0.001), except on day two (Figure 1).

#### Pyruvate and lactate

**Figure 2:**
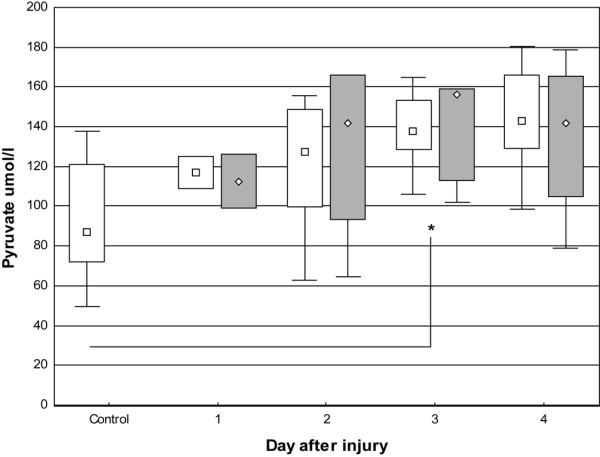
Box-and-whisker plots showing median (interquartile) pyruvate concentrations in microdialysate from days one to four. Open boxes indicate uninjured skin and controls; shaded boxes indicate burned skin. Controls, *n *= 9. Uninjured skin on day 1, *n *= 2; day 2, *n *= 5; day 3, *n *= 6; and day 4, *n *= 5. Burned skin on day 1, *n *= 2; day 2, *n *= 5; day 3, *n *= 6; and day 4, *n *= 4. *P < 0.05.

**Figure 3:**
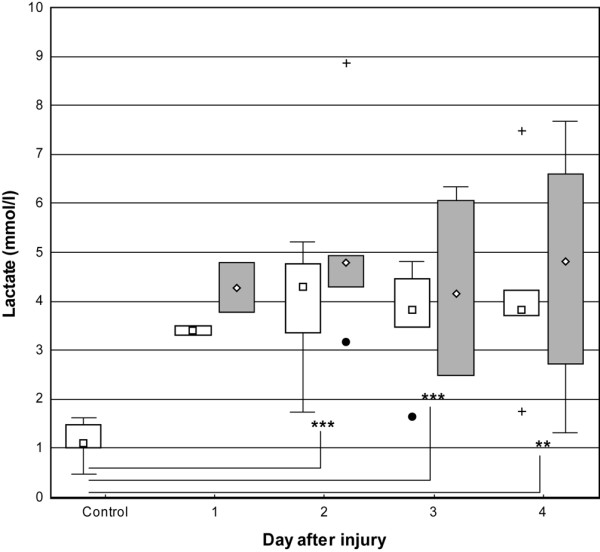
Box-and-whisker plots showing median (interquartile) concentrations of lactate in microdialysate from days one to four. Open boxes indicate uninjured skin and controls; shaded boxes indicate burned skin. Controls, *n *= 9. Uninjured skin on day 1, *n *= 2; day 2, *n *= 5; day 3, *n *= 6; and day 4, *n *= 5. Burned skin on day 1, *n *= 2; day 2, *n *= 5; day 3, *n *= 6; and day 4, *n *= 4. **P < 0.01 and ***P < 0.001. Filled circles and plus signs indicate outliers and extremes, respectively.

The pyruvate concentration in skin showed a tendency to increase, but to a lesser extent than lactate, during all study days in patients in uninjured skin (131 mmol/l [63 to 181]) and burned skin (142 mmol/l [65 to 179]) compared with controls (87 mmol/l [49 to 138]). This reached significance at day three (*p *< 0.05). The lactate concentration in skin of controls was within the reference range (1.1 mmol/l [0.5 to 1.6]), whereas lactate concentration in burned patients was significantly (*p *< 0.01) higher in uninjured and burned skin (3.8 mmol/l [1.6 to 7.5] and 4.6 mmol/l [1.3 to 8.9], respectively). There was a tendency for higher median values in burned skin than in uninjured skin (Figures 2 and 3). There was a significant correlation (*p *< 0.05) between glucose and pyruvate (*r *= 0.54) as well as lactate (*r *= 0.64).

#### Tissue lactate/pyruvate ratio

**Figure 4:**
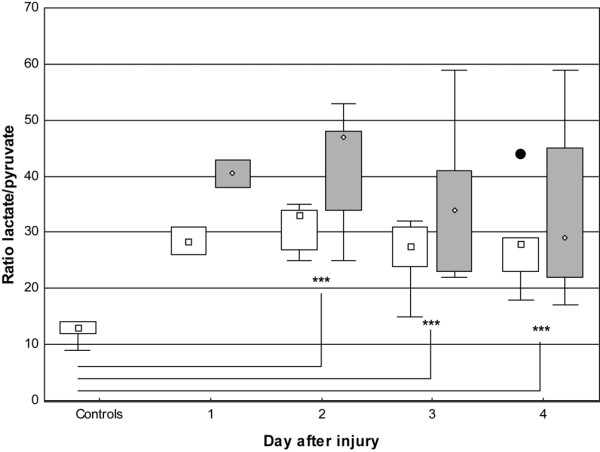
Box-and-whisker plots showing lactate/pyruvate ratio in microdialysate from days one to four. Open boxes indicate uninjured skin and controls; shaded boxes indicate burned skin. Controls, *n *= 9. Uninjured skin on day 1, *n *= 2; day 2, *n *= 5; day 3, *n *= 6; and day 4, *n *= 5. Burned skin on day 1, *n *= 2; day 2, *n *= 5; day 3, *n *= 6; and day 4, *n *= 4. ***P < 0.001. Filled circle indicates outlier.

The tissue lactate/pyruvate ratio increased two- to fourfold, with significantly higher values in burned patients during study days two to four. The ratios in uninjured skin were 33 (day two), 27.5 (day three), and 28 (day four) and in burned skin 47 (day two), 34 (day three), and 29 (day four); the ratio in controls was 13 (*p *< 0.001). There was a peak on day two in both uninjured and burned skin, but the ratio in the uninjured skin then returned to the initial value on day three and remained stable thereafter. The burned skin was slower to recover and reached the initial value on day four (Figure 4). There was no correlation between glucose and the lactate/pyruvate quotient (*r *= 0.13).

#### Glycerol

**Figure 5:**
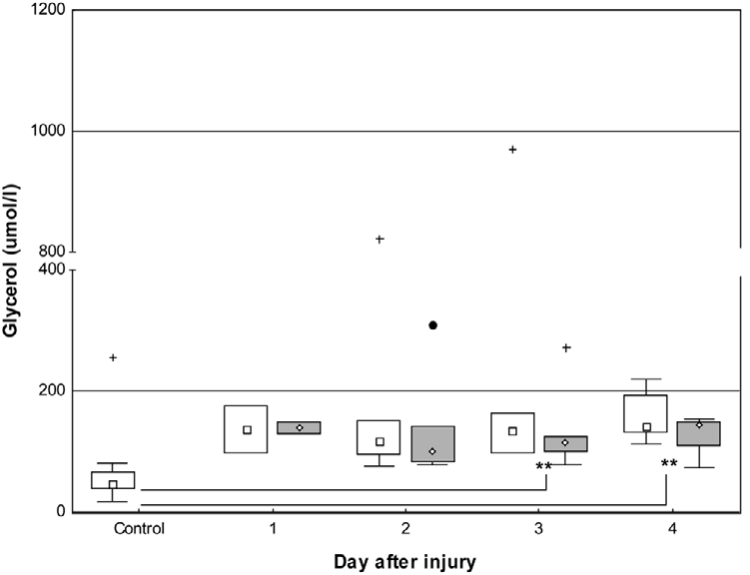
Box-and-whisker plots showing median (interquartile) concentrations of glycerol in microdialysate from days one to four. Open boxes indicate uninjured skin and controls; shaded boxes indicate burned skin. Controls, *n *= 9. Uninjured skin on day 1, *n *= 2; day 2, *n *= 5; day 3, *n *= 6; and day 4, *n *= 5. Burned skin on day 1, *n *= 2; day 2, *n *= 5; day 3, *n *= 6; and day 4, *n *= 4. **P < 0.01. Filled circle and plus signs indicate outlier and extremes, respectively.

The concentration of glycerol in the skin was increased in the patient group during the study period controls 46.4 μmol/l [17.1 to 257.3], burned skin 136.6 μmol/l [75.9 to 970], and uninjured skin 123.4 μmol/l [73.4 to 309]) and this reached significance on days 3 and 4 (*p *< 0.01) (Figure 5).

#### Urea

**Figure 6:**
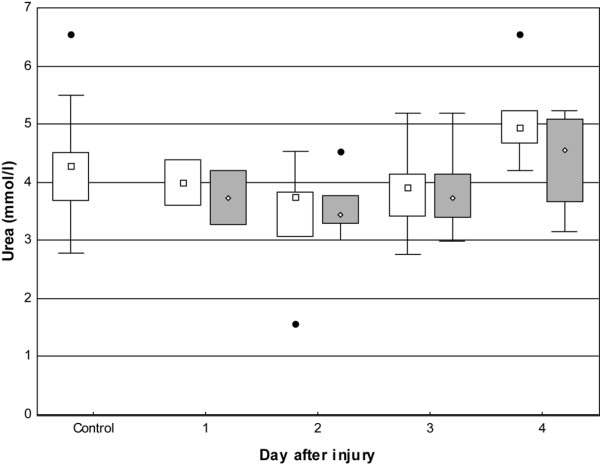
Box-and-whisker plots showing urea in microdialysate from days one to four. Open boxes indicate uninjured skin and controls; shaded boxes indicate burned skin. Controls, *n *= 9. Uninjured skin on day 1, *n *= 2; day 2, *n *= 5; day 3, *n *= 6; and day 4, *n *= 5. Burned skin on day 1, *n *= 2; day 2, *n *= 5; day 3, *n *= 6; and day 4, *n *= 4. No significant differences were noted compared with controls. Filled circles indicate outliers.

The concentration of urea was within the reference range in controls (4.6 mmol/l [1.1 to 7.6]) as well as in uninjured skin (4.1 mmol/l [1.6 to 6.5]) and injured skin (3.5 mmol/l [3.0 to 5.2]) (Figure 6).

## Discussion

The main findings of this study were that microdialysis could be applied to critically ill burned patients and that skin metabolic processes could be followed for several days. This technique seems to picture events in the skin, which are not recognised in the central circulation. There is acidosis in the skin. The systemic effects of trauma on the homeostasis of glucose and fat were also illustrated by the technique and showed hyperglycaemia and lipolysis.

We do recognise the limitation of this study in the small number of patients and the heterogeneity in the burn trauma (chemical/flame injury). In regard to the type of trauma, the chemical burns were considered representative of a significant tissue trauma because the fluid needs and the clinical course were rather similar to those of the flame burns.

### Lactate and pyruvate

The production of lactate and pyruvate in the dermis has been investigated in healthy humans, and the values of lactate in dermis have been shown to be higher than those in plasma [22]. It is also recognised that hyperglycaemia increases the production of lactate in subcutaneous tissue by up to 50% [29]. As anticipated, we found a correlation between increased tissue glucose levels and increases in both tissue lactate and pyruvate levels.

During non-ischaemic conditions, a parallel increase in pyruvate is seen, and the ratio of lactate to pyruvate remains stable. An increased ratio indicates ischaemia [22,30]. Indeed, increased lactate/pyruvate ratio is considered a sensitive marker of ischaemia.

The ratio was significantly higher in patients in both burned and uninjured skin, during early as well as late resuscitation. This suggests ischaemia of the skin, despite the lack of signs of acidosis in the central circulation. The difference between uninjured and burned skin was not significant, but there was a trend toward higher values in burned skin. This increased ratio eventually returned to the value of the uninjured skin on day four, which may be explained by an initial relative under-resuscitation and hypovolaemia that affected the circulation in the skin, as suggested in several studies using other endpoints for resuscitation [7,13]. Haemodilution is often seen as a consequence of the aggressive early fluid resuscitation in burns, and this adds a further reduction in oxygen delivery and a deposition of fluid in the injured tissue, both of which may lead to reduced perfusion and tissue ischaemia [18,31].

In patients with severe burns, the systemic inflammatory response syndrome is present [2]. Disseminated intravascular coagulation with loss of platelets is due to thromboses in the microvasculature, which results in an impaired microcirculation that could lead to an increased lactate/pyruvate quotient, and thereby another plausible explanation for our findings is added.

We found a clear increase in the lactate/pyruvate quotient, especially during days three and four postburn. Since the time of the study, new knowledge on unwanted degradation of pyruvate during storage at -20°C (that is, a 7% to 10% pyruvate decrease per month) has been revealed (application note 8, 2006, CMA Microdialysis AB). It needs to be stressed that this was not known at the time of the study. Given the storage times of the present study, there is a risk for reporting a falsely high lactate/pyruvate ratio. Recalculating the ratios, assuming a three month analysis delay, the ratios are still more then twice those of the controls. We would also like to stress that, at the time of the study, all procedures were handled according to manufacturers' recommendations.

The patients in this study received what appear to be excessive amounts of fluids, leading to urinary output levels above the endpoint goals. This may be explained in part by the sizes of the burn injuries as stated above. All the patients in the present study had major burns (median TBSA percentage, 59%), but it needs to be pointed out that the study is in line with modern fluid treatment traditions, which at present tend to use larger resuscitation volumes [15].

### Glucose

Microdialysis has been described most fully for glucose studies and is known to accurately reflect changes in both blood and tissue concentrations [22,32]. In this study, blood glucose concentration was influenced by trauma-induced insulin resistance, which peaked on day two and then gradually decreased to a lower but still increased value. It is important to underline that this study was conducted prior to the era of tight glucose control, but due to the risk of hypoglycaemia [33], insulin treatment is still not standard during the early resuscitation period in our unit although we do use an aggressive glucose control protocol thereafter [6,34,35]. Glucose concentration in the skin initially followed the same pattern as in blood but surprisingly continued to increase throughout the study period in both burned and uninjured skin. It seems that insulin resistance continues locally, in this case in the dermis, even when the systemic situation is beginning to normalise.

The reason for the trauma-induced insulin resistance is not fully understood, but increasing evidence in skeletal muscle indicates a low interstitial insulin concentration, making the capillary wall rate-limiting and capillary recruitment essential for optimised insulin kinetics in peripheral tissue [36,37]. Such an explanation would also be relevant for burns given that microvascular disturbances during early resuscitation for shock are to be expected. What then becomes puzzling is that experimental studies either on induced vasoconstriction or ischaemia all show decreased glucose levels interstitially [26].

There is also increasing evidence that an impairment of cellular metabolism (that is, mitochondrial dysfunction resulting in bio-energetic failure [38] rather than hypoperfusion and concomitant tissue hypoxia) is the reason for organ dysfunction secondary to trauma and sepsis [39-41]. The microdialysis result of this study mimics the result of several such studies examining the cellular metabolism in sepsis. We do lack signs of hypoperfusion (that is, systemic acidosis) and the patients were fully resuscitated and likely to be hyperdynamic in their circulation. Still, there is local acidosis in skin (increased lactate/pyruvate quotients) but local skin hyperglycaemia indicates sufficient blood flow for glucose delivery. But at the same time, the use of the glucose seems impaired, which could be explained by cytopathic hypoxia. It is of particular interest that these cellular responses have not yet been described in association to burn chock resuscitation. Furthermore, it is well known that hyperglycaemia worsens brain oedema after injury [42]. It might therefore be argued that the finding of increased glucose levels interstitially, in combination with the permeability increase and negative imbibition pressures (both well known factors for burn oedema formation), may further promote burn-induced skin oedema.

### Urea

Urea is an often-used endogenous reference substance *in vitro*. Other authors [26,29] have proposed the hypothesis that comparing the recovery (that is, the fraction of the absolute tissue concentration recovered in the perfusate) values of urea *in vivo *and the substance of interest would assess the relative *in vivo *recovery of the given substance. We found median recovery values of 80% and 74% for glucose in uninjured and burned skin, respectively, and 96% to 100% for urea for all categories (controls and injured and uninjured skin), and this strongly supports the view that our microdialysis results accurately reflect events in tissues.

### Glycerol

Catecholaminergic stimulation of fat cells is a key mechanism in the regulation of fat metabolism in patients with burns[43,44]. Lipolysis causes weight loss and affects outcome, and this is a serious problem in burn care [6]. It has been recognised since the 1980s that agents that block beta-adrenergic receptors improve outcome by reducing tissue catabolism and, in particular, lipolysis [43]. Microdialysis has been used in numerous studies of lipolysis, showing increased concentrations of glycerol in subcutaneous adipose and muscle tissue. Sympathetic stimulation effectively inhibits the antilipolytic effect of insulin by inducing insulin resistance [45]. It is also important to note that lipolysis differs among different tissues [45]. We have shown three- to fourfold increases in the concentrations in the skin of burned patients. Our belief is that the observed increase in glycerol reflects the lipolysis induced by the trauma stress response. Similar effects have been shown experimentally in humans during induced sympathetic stimulation or euglycaemic-hyperinsulinemic clamps [45]. The finding may have implications in studies of lipolysis, making it possible to study intervention (for example, beta blockade or insulin) in different tissues.

## Conclusion

Microdialysis can monitor effects induced by burns on tissue lactate, pyruvate, glucose, urea, and glycerol for several days. It seems to picture events in the skin which are not found in the central circulation. There seems to be acidosis in the skin, which might be related to ischaemia secondary to the fluid resuscitation. These findings are also consistent with cytopathic hypoxia and reflect metabolic cell dysfunction. To our knowledge, this response (previously described in sepsis) has not been described in burn injuries before. Furthermore, the impaired glucose metabolism may create an osmotic gradient from blood, which may further contribute to the oedema formation. All these factors may affect skin survival, with risk for deepening of injury and the need for further surgery. Another interesting finding is that lipolysis can be monitored successfully.

## Key messages

• Our results show that, despite current standard regimens for resuscitation of burns, there is local acidosis in skin. This is most likely due to impaired cell metabolism, but local ischaemia cannot be excluded.

• Microdialysis offers a possibility to monitor tissue effects in the development of new resuscitation formulas and evaluate future pharmacological interventions aiming at cell metabolism.

• Glucose and fat metabolisms differ locally from systemic values, and microdialysis may thus be used to better understand the peripheral pathophysiology of, for example, glucose intolerance.

## Abbreviations

MAP = mean arterial pressure; TBSA = total body surface area burned.

## Competing interests

The authors declare that they have no competing interests.

## Authors' contributions

AS and FS contributed in all phases of the study and the preparation of the manuscript. IS substantially contributed to acquisition, analysis, and interpretation of data. All authors read and approved the final manuscript.

## Figures and Tables

**Table 1:** Patient and control data

	Patients *n *= 6	Controls *n *= 9
Age in years^a^	27.5 (17–31)	29 (22–42)
TBSA percentage^a^	59 (33.5–90)	-
Female/Male	1/5	4/5
Survival	6	-
Inhalation injury	1	-
Days in hospital^a^	60.5 (86)	-

^a^Data are presented as median (range). There are no significant differences between the groups. TBSA, total body surface area burned.
